# Design and Development of Magnetostrictive Actuators and Sensors for Structural Health Monitoring

**DOI:** 10.3390/s20030711

**Published:** 2020-01-28

**Authors:** Jamin Daniel Selvakumar Vincent, Michelle Rodrigues, Zhaoyuan Leong, Nicola A. Morley

**Affiliations:** Department of Materials Science and Engineering, University of Sheffield, Sheffield S1 3JD, UK; jdsvincent1@sheffield.ac.uk (J.D.S.V.); mrodrigues2@sheffield.ac.uk (M.R.); z.leong@sheffield.ac.uk (Z.L.)

**Keywords:** structural health monitoring, composites, magnetostrictive actuator, sensors, data acquisition system

## Abstract

Carbon Fibre Reinforced Polymer composite (CFRP) is widely used in the aerospace industry, but is prone to delamination, which is a major causes of failure. Structural Health Monitoring (SHM) systems need to be developed to determine the damage occurring within it. Our motivation is to design cost-effective new sensors and a data acquisition system for magnetostrictive structural health monitoring of aerospace composites using a simple RLC circuit. The developed system is tested on magnetostrictive FeSiB and CoSiB actuator ribbons using a bending rig. Our results show detectable sensitivity of inductors as low as 0.6 μH for a bending rig radii between 600 to 300 mm (equivalent to 0.8 to 1.7 mStrain), which show a strain sensitivity resolution of 0.01 μStrain (surface area: ~36 mm^2^). This value is at the detectability limit of our fabricated system. The best resolution (1.86 μStrain) was obtained from a 70-turn copper (~64 μH) wire inductor (surface area: ~400 mm^2^) that was paired with a FeSiB actuator.

## 1. Introduction

Due to their high strength-to-weight ratio, carbon-fibre reinforced polymer composites (CFRPs) are widely replacing the metals traditionally used in the aviation industry. The superior properties of CFRPs are attributed to the layered structures of the composites which combine the best of its constituent materials. However, these complex structures are prone to damage like matrix cracking, fibre pull-out, and delamination [1]. Composite structures used in aircrafts and helicopters can suffer from serious degradation due to aerodynamic loading, exposure to harsh environment, cyclic loading, sudden impacts, operator abuse, and negligence. These damages can lead to safety concerns and unplanned maintenance [2]. 

These damages, whether visible or barely visible, can significantly reduce the strength of the material [3]. In these circumstances, structural health monitoring (SHM) systems need to be implemented to effectively detect and monitor damage. The use of such a system allows the remaining service life of the material to be efficiently predicted prior to failure. If the detected damage is serious, emergency maintenance can be performed before catastrophic failure [1]. Fibre optic sensors, piezo-electric sensors, electrical strain gauges, acoustics and ultrasonic sensors are major SHM techniques which are currently being investigated to detect damage in composites [2]. Existing SHM technologies such as ultrasonic wave detection system, acoustic wave propagation, vibration based SHM etc. are used to detect cracks and delamination in composites, but have proven to be unreliable as they are incapable of crack detection at the micro level and are triggered only after a serious damage has occurred. However, they are still in use due to the lack of an alternative SHM technology which can overcome these drawbacks [4,5,6]. The aftermath of major damage detection (delamination, impact damage, fatigue, etc.) in critical components (airframe or jet engine), involves safety issues, maintenance costs and operational disruption [7]. Other notable disadvantages of the existing SHM methods are: they are expensive, difficult to install, have complex geometries, require lamb-wave generation, relies only on a computer without cross verification, as well as require large battery backup to operate [8,9].

One alternative to this method is to incorporate magnetostrictive materials in a sensor-actuator setup [10]. Magnetostriction is the property of ferromagnetic materials, whereby when they are placed in a magnetic field, they experience a change in their physical dimensions. This effect is attributed to the coupling of magnetic and elastic forces. Depending on the external magnetic field, the material can either expand or shrink in the direction of the field [11,12]. The inverse of this effect known as the Villari effect, which means the magnetisation of the material changes as a function of an applied stress [13]. The ability of magnetostrictive materials to experience these changes make them good candidates for their use as sensors and actuators [14]. In the sensor-actuator setup, the actuators are magnetostrictive materials which are embedded into the CFRP. This can be either in the form of particles [15], wires [16] or ribbons [10]. Actuators in the form of magnetostrictive wires made up of Fe_78_Si_7_B_15_ and Co_78_Si_7_B_15_ embedded over CFRP have been subjected to tensile loading to study damage detection [16]. Terfenol-D, a highly non-linear material has been commonly used as actuator in most of the previous work due to its large magnetostriction [15,17,18]. However, a major disadvantage of using Terfenol-D is its cost-effectiveness. Since the SHM system will be applied to a large surface area of the aircraft, it is important to consider the cost and weight of the materials when selecting the actuator. Cobalt-based and ferrous-based magnetostrictive materials are some of the available cost-effective options, which allow easy fabrication due to their strength, ductility and easy workability [19]. The combined use of magnetostrictive CoSiB ribbons as an actuator and a suitable magnetic sensor was seen to enable damage detection in composites [10]. Magnetostrictive actuators in the form of particles have been embedded over a CFRP cantilever beam with a sensing coil wound around it to detect delamination [20,21]. However, the use of particles as actuators can cause agglomeration, which inhibits the mechanical properties of CFRP thereby inducing delamination [22]. Also, it is not practically possible to wind coils around an entire aircraft. Recent research has proved that the use of magnetostrictive ribbons as actuators can give high quality results as they possess excellent magneto-mechanical properties [10]. Furthermore, FeSiB ribbons can be magnetised and demagnetised easily [23]. Iron has better magnetostrictive properties when compared to cobalt [24]. Due to their amorphous structure they show excellent ductility, high tensile strength, resistance to corrosion, low eddy current loss, and low hysteresis loss [25,26]. Considering the above properties, ferrous-based and cobalt-based magnetostrictive ribbons are chosen to be used as actuators for sensing delamination. 

The sensor to be used in conjunction with the actuator is designed using the principle of an inductance coil. In most of the previous work, the sensors developed are a combination of an excitation coil and a sensing coil, which are used for detecting crack propagation [17,20,27,28]. The working principle of induction coils is electromagnetic inductance [29]. In general, they can be either wound around a magnetic core or left without a core (i.e., air core). Air core sensors represent an inductance coil wound on a non-ferromagnetic material such as glass, plastic etc. [30]. The inductance coil in an air cored sensor ranges from a single loop coil to multi loop coils. Air cored sensors are beneficial over ferrite cored sensors as the losses arising from magnetic hysteresis and eddy currents are lower [29]. In some cases, the excitation coil and sensing coil are wound over a U-shaped magnetic core where the excitation coil acts as an electromagnet which excites the atoms in the actuator. This induces an open circuit voltage across the sensing coil. When strain is applied over the material, the sensing coil picks up the change in root mean square (RMS) value of the voltage, which corresponds to the damage occurring in the material [27].

By using magnetostrictive actuators along with a sparse array of inductance sensors, an effective damage detection system can be developed for structural health monitoring in composites. The benefit of using a sparse array as compared to a compact array is that it is capable of covering a larger area. This in turn implies that the use of fewer sensors for the same area which in turn leads to lower overall weight and reduces the need for complex connections. Finally, any localised damage occurring in a composite can be investigated from different angles.

## 2. RLC Circuit for Damage Sensing

**Figure 1:**
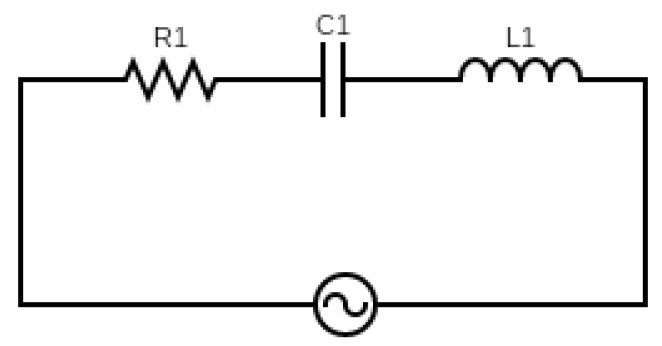
A series RLC circuit with an AC source, where R1 is a resistor, C1 is a capacitor and L1 is an inductor.

For the damage sensing system, an RLC circuit was developed. Its main advantage is that it is easy to construct thereby massively reducing the requirement of complex circuitry. This in turn makes the overall sensing system less bulky, light and easy to operate. The number of components involved are few, which contributes to making it a very cost-effective system when considered for large scale manufacturing. Another notable advantage of using an RLC circuit is its high tunability [31]. Figure 1 shows a typical series RLC circuit driven by an AC source, such as a function generator. Capacitors (C1) and inductors (L1) are energy storage components, where the former stores electrical energy and the latter, magnetic energy. In the absence of a resistor, the RLC circuit becomes an LC oscillator circuit, where the electromagnetic energy of the circuit is constant. When a significant resistance is present, as in the case of RLC circuits, the electromagnetic energy diminishes with time. This is on account of its dissipation in the form of heat through the resistor. This means the charge on the capacitor and the current in the inductor oscillate and tend towards zero with time, which leads to a decaying, transient response of the RLC circuit with time.

Also, the RLC circuit is analogous in behaviour to a one-dimensional spring-mass system, which in turn represents a damped harmonic oscillator. The solution of the equation describing the RLC circuit is thereby given as:

(1) $$q=Q_{m}e^{\frac{-t}{\varsigma }}\;cos\left(\omega_{d}t+\phi \right)$$

where q is the charge on the capacitor plates, $e^{\frac{-t}{\varsigma }}$ depicts the exponentially decreasing amplitude of the oscillations, $\omega_{d}=\sqrt{\frac{1}{LC}-{\left(\frac{R}{2L}\right)}^{2}}=\frac{1}{2L}\sqrt{\left[\frac{4L}{C}-{\left(R\right)}^{2}\right]}$ depicts the damped frequency where *L* is the inductance, *C* is the capacitance and *R* is the resistance. The cosine term depicts oscillation of the charge with respect to time.

Depending on the value of the resistor, the RLC circuit can experience either an underdamped, overdamped or critically damped response. Therefore, (i) if $R≻\sqrt{\frac{4L}{C}},$ the circuit is overdamped, (ii) if $R<\sqrt{\frac{4L}{C}},$ the circuit is underdamped, and (iii) if $R=\sqrt{\frac{4L}{C}},$ the circuit is Critically damped [31]. In the underdamped case, the charge oscillates with an amplitude that exponentially decays to zero with time. In the overdamped case, the charge experiences no oscillation and approaches zero in a monotonic fashion. When the reactance of the inductor and the capacitor becomes equal to each other, the circuit is said to resonate at a resonance frequency (*f*) given by:

(2) $$f=\frac{1}{2\pi \sqrt{LC}}$$

where *L* is the inductance and *C* is the capacitance^28^. As long as the system remains underdamped, the unknown inductance of any connected inductance can be determined as long as the value of the other components are known.

## 3. Materials and Methods

### 3.1. Characterisation of RLC Circuit

In order to develop the sensing system, it is vital to characterise the circuit for different combinations of inductor and capacitor values with an aim to operate the circuit in the underdamped regime. This is because the circuit is capable of an oscillatory transient response only in this setup. This preliminary step helps to down select the various combinations to a few working combinations that can be used in the final design. 

To fabricate the sensors, plastic M3 washers were chosen as they provide a frame for easy winding of the copper coils. Copper wire of diameter 0.224 mm was wound around the washers in a clockwise direction to fabricate pancake inductance sensors with 30, 50 and 70 turns as shown in Table 1. 

**Figure 2:**
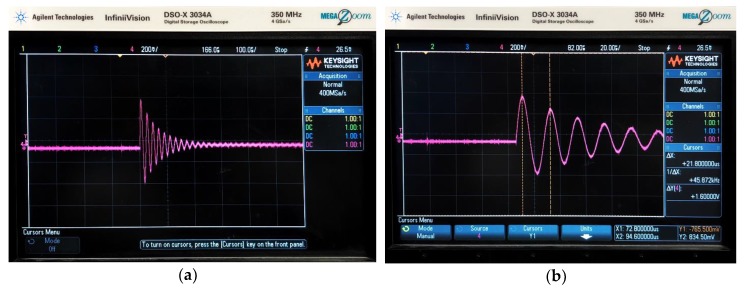
(**a**) Oscilloscope output for an undamped transient response of an RLC circuit where *L* = 64.3 μH and *C* = 0.2 μF with time on the x-axis and charge across the capacitor along the y-axis. (**b**) The cursors represent time period for the oscillation with the highest amplitude, the inverse of which gives us the resonance frequency.

Four different capacitors with capacitances 0.1, 1, 10 and 100 μF along with the fabricated sensors were primarily chosen for developing the data acquisition system. The capacitors were used in pairs connected in parallel to give usable equivalent capacitance values of 0.2, 2, 20 and 200 μF. In addition to the fabricated sensors, a Farnell air-cored sensor of inductance 0.6 μH was also considered. Each equivalent capacitor was paired with each inductor and the response (whether overdamped or underdamped) was theoretically calculated as per the conditions mentioned above. The AC input to the circuit was driven by a function generator in the form of a square wave with a frequency of 1 kHz and 5 V peak-to-peak voltage. The output was visualised on a digital oscilloscope as seen in Figure 2 for the various inductor and capacitor combinations.

**Figure 3:**
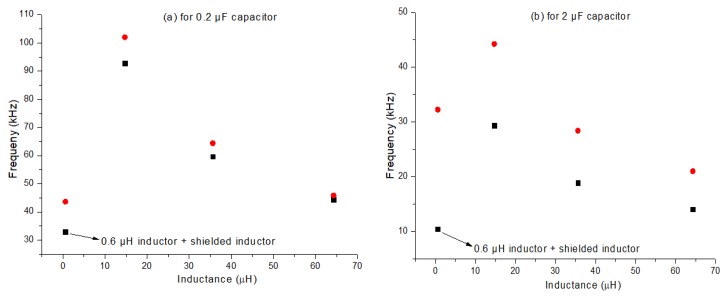
(**a**) Resonance frequency of the RLC circuit using 0.2 μF capacitors. (**b**) Shows the Resonance frequency of the RLC circuit using 2 μF capacitors. In both the figure the red dots represent the experimentally derived and the black dots represent the theoretically calculated resonance frequencies.

From the calculations, it was observed that the 0.2 and 2 μF capacitor gave an underdamped response for all inductor values except the store-bought 0.6 μH Farnell inductor. Whereas, the 200 μF capacitor gave an overdamped response for all inductor values. The 20 μF capacitor gave an overdamped response for all inductor values except the 64.3 μH inductor (70 turns). In order to make the circuit workable for the off-the-shelf 0.6 μH Farnell inductor with the capacitors 0.2 and 2 μF, a shielded inductor of inductance 115.7 μH was added in series with it. This increased the overall inductance of the circuit and changed the response from overdamped to underdamped making it possible to observe a response at an inductance value of 0.6 μH. The frequency responses for the various inductor combinations with the 0.2 and 2 μF capacitors from the oscilloscope were compared with the theoretically calculated frequency using equation [2]. Figure 3 represents the deviation in the theoretically calculated and experimentally derived resonance frequencies for the 0.2 and 2 μF capacitors. In Figure 3a it can be observed that at higher frequency there is a larger deviation between the experimental and theoretical values. This can be explained with the help of equation [2]. From which it can be understood that frequency and inductance are inversely proportional. Which means the measurement error from the inductor will also decrease at lower frequency. This explains the reason for lower percentage error at lower frequency. In Figure 3b it can be observed that for a 2 µF capacitor the difference between experimental and theoretical values are much larger when compared to 0.2 µF capacitor. This is due to tolerance factor. When the capacitors were measured with an RLC meter 0.2 µF capacitor gave an actual capacitance value of 0.185 µF and 2 µF gave a value of 1.402 µF. The percentage difference between the measure values and nominal value (marked value) is 7.5% and 29.9% because of which 2 µF capacitor gives larger difference. As the strain sensitivity of the 0.2 µF capacitor is higher than that of the 2 µF capacitor. However, this result is not enough to rule out the possibility of using 2 μF capacitor and the bending rig test will be discussed using both the 0.2 and the 2 μF capacitor.

### 3.2. Inductance Sensors and Data Acquisition

**Figure 4:**
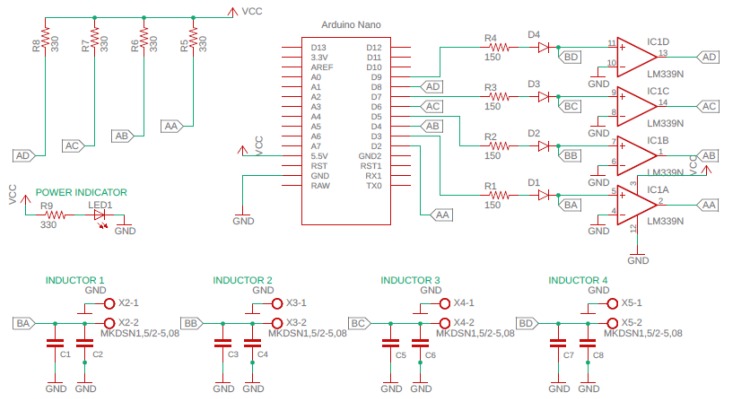
Circuit diagram for the data acquisition system with four inputs.

Using the methodology from the previous section, a simple system can be developed for magnetostrictive SHM of composites. An Arduino Nano (Cool Components Ltd., Stockbridge, UK) with the ATMEGA328 ADC processor is selected as the microcontroller for this system. A quad comparator LM339 is attached to the Arduino, which converts an analogue signal into a digital signal. The LM339 is chosen as it is faster than a typical operational amplifier (op-amp; a typical op-amp has a slew rate of 11 V/µS whereas, the LM339 has a slew rate of 0.5 V/µS). In addition, op-amps take more time to recover from saturation since they operate at a linear frequency with negative feedback. Also, when high frequency signals are involved, they endure delays in the order of tens of microseconds. Hence, the LM339 is widely used when two output voltages have to be compared. LM339 is a 14 pin IC, which can take four input signals and can give four outputs simultaneously. Since the LM339 can input four signals, it enables the system to procure data from four sensors concomitantly. This helps to monitor crack growth more precisely. The designed circuit diagram for such a data acquisition system is illustrated in Figure 4. The Arduino’s built-in comparator cannot be used as (1) Its clock speed is insufficient to determine small changes in inductance, and (2) It cannot be used to pick up more than one signal. A multiplexer may be used to overcome (2) but will likely introduce additional noise to the system, and is also more expensive than the LM339.

When the voltage in circuit turns positive, the LM339 begins to float, and thus requires a pull up resistor. When the circuit becomes negative, the LM339 outputs to ground. When a 5 V pulse is applied from the Arduino pin 13, the comparator will generate a square wave.

**Figure 5:**
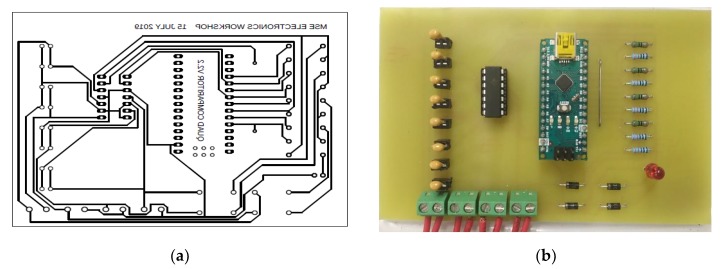
(**a**) Design of PCB (**b**) Fabricated PCB

From the resonant frequency Equation (2), the inductance in the sensor depends on the capacitance in the data acquisition system. Hence, it is necessary to choose the correct capacitor value to make the sensor more sensitive to detect even a small change in inductance due to the magnetostriction from the actuator. Sensitivity variation in the sensor due to the change in capacitance is discussed in the below sections. Using the design shown in Figure 4, the printed circuit board (PCB) was designed using Autodesk AutoCAD which is shown in Figure 5a, b shows the fabricated data acquisition system.

### 3.3. Sample Fabrication

Amorphous Fe_78_Si_7_B_15_ and Co_78_Si_7_B_15_ ribbons fabricated by arc-melting followed by rapid injection on to a water-cooled wheel supplied by Vacuumschmelze were selected as the actuator materials. Twill weave carbon fibre prepreg VTC-401 supplied by SHD Composites Ltd. was employed for sample fabrication. Using the technique of vacuum bagging, composite laminates with dimensions of 170 × 120 × 4.5 mm were fabricated for the trial bending tests. Magnetostrictive ribbons made of Fe_78_Si_7_B_15_ and Co_78_Si_7_B_15_ were placed along the middle ply as well as on the top surface of the composite laminates. Only one ribbon is used, in comparison to previous studies [32]. The laminate was sealed under vacuum at -0.04 bar and later maintained in an autoclave at 6 bar pressure. The curing cycle was initialised with a temperature ramp rate of 3 °C/min until the composite reached 60 °C. Following this it was held at 60 °C for 60 min. Again, the temperature was ramped up till 120 °C at the rate of 3 °C/min. It was then held at 120 °C for 60 min after which it was cooled down to room temperature. The composite laminate was cut into the desired sample dimensions of 150 × 25 × 4.5 mm using a tile cutter.

## 4. Experimental Testing

### 4.1. SHM Schema

**Figure 6:**
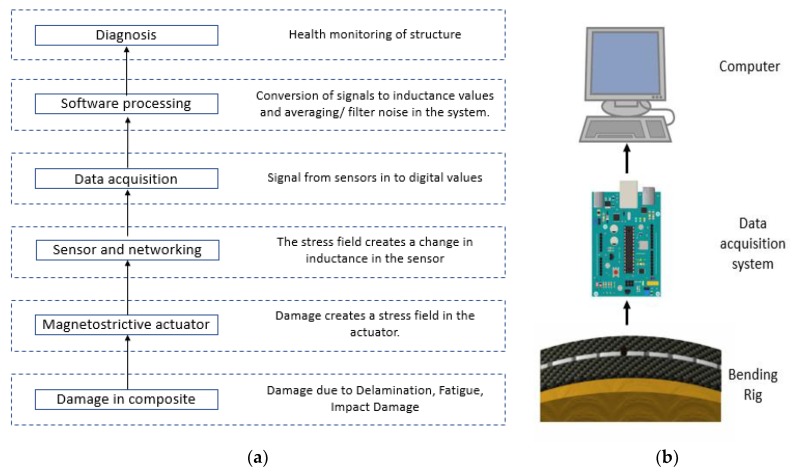
(**a**) Functional block diagram of the system (**b**) Experimental setup for bending rig test.

When damage occurs in a composite embedded with magnetostrictive actuator, due to inverse magnetostriction, i.e., the Villari effect, the actuator undergoes an increase in its magnetisation. This interacts with the magnetic field present around the inductor, which creates an induced voltage in the circuit seen as a change in inductance in the sensor. The change in inductance changes the resonant frequency of the data acquisition system, which is recorded by the Arduino. The data acquisition system converts the output signal from the sensors to a digital form, which can be further processed. Post-processing the data improves the signal-to-noise ratio and generates user-readable inductance values. Hence, in a sparse array of sensors, if any sensor experiences a change in its inductance, this sensor can be identified via the change. From this identification, it can be asserted that damage has occurred in that region of the composite. Figure 6b depicts the functional block diagram with the various steps involved in the bending rig test.

### 4.2. Experimental Setup

**Figure 7:**
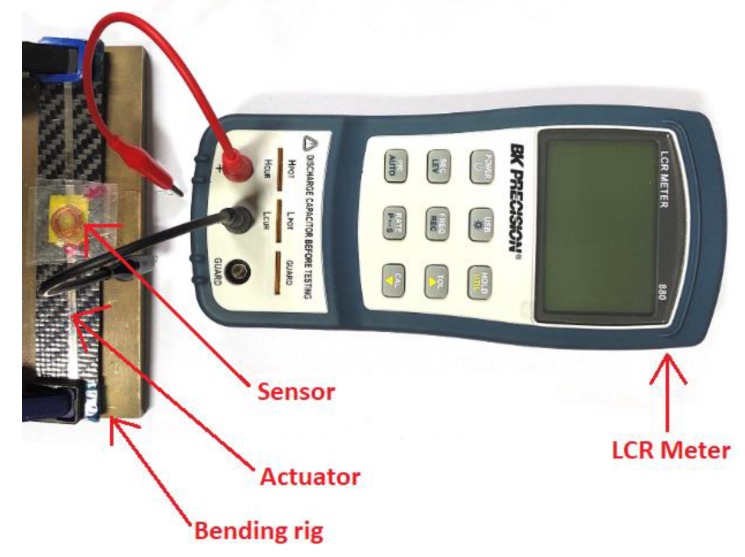
Setup of bending rig test with RLC meter with the main components labelled.

The fabricated samples were tested using bending rigs of radii 300, 400, 500 and 600 mm. These correspond to strain values of 0.83, 0.98, 1.24, and 1.66 mStrain (10^−3^ x strain), respectively. A sensor was fixed to the middle of the sample above the actuator ribbon. When stress is applied to the sample, it creates deformation within the sample. This creates a change in magnetization in the actuator, which in turn changes the inductance in the sensor, which is recorded by the data acquisition system. The change in inductance with respect to different bending rig radii and with different capacitance values was obtained. The obtained data can be plotted as a function of time for loading and unloading. These values were obtained using BK precision RLC meter as shown in Figure 7. The test was conducted in a Faraday cage to prevent the influence of any external magnetic field.

## 5. Results and Discussion

### 5.1. Validating Fabricated Sensor Electronics

**Figure 8:**
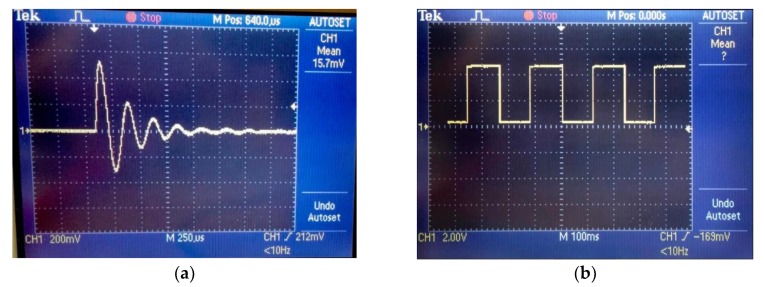
(**a**) RLC response (**b**) Comparator output.

Following the fabrication of the data acquisition system, the output signal from it was observed using an oscilloscope. Figure 8a shows the RLC transient response of the circuit. The graph shows damped oscillations of charge with respect to time which is typical for an underdamped RLC circuit. Figure 8b shows the square wave output from the comparator.

The oscilloscope results were similar to those obtained from the preliminary results as in Figure 2. This suggests that the fabricated data acquisition system was in good working order. When a ferromagnet was brought in proximity of the sensor, a change was observed in the output of the sensors. On the oscilloscope, this was visualised as a decrease in the amplitude and time period of the underdamped oscillations, thereby increasing the resonant frequency. Bringing the magnet close to the sensor changes the magnetic field around the sensor which causes the eddy currents generated to decrease the inductance of the sensor. This decrease in inductance is seen in the output of the system as a decrease in the time period of the damped oscillations.

### 5.2. Change in Inductance During Loading and Unloading

**Figure 9:**
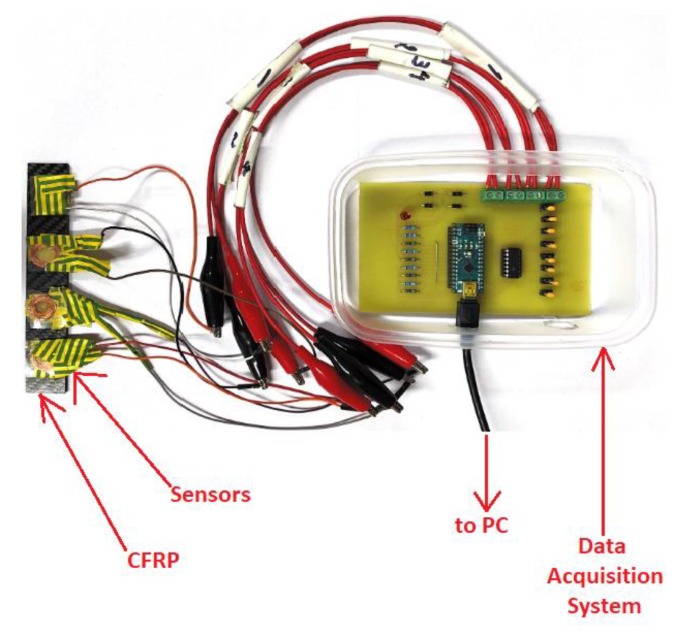
CFRP with four sensors on the top surface connected to the fabricated data acquisition system.

**Figure 10:**
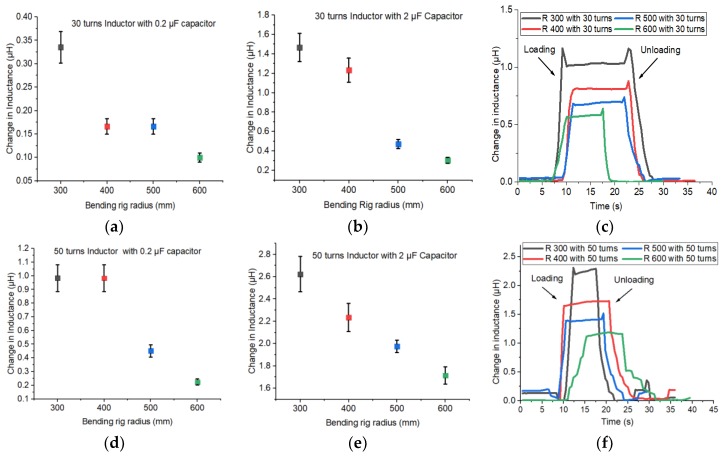

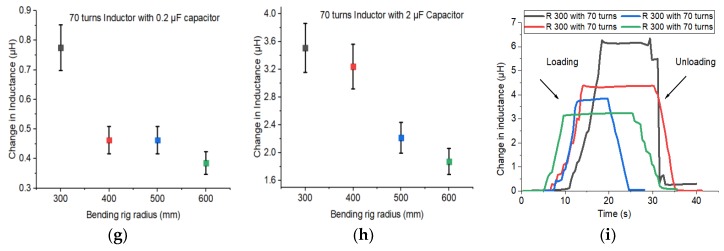
(**a**–**i**) Change in inductance results of the bending rig test for composite with CoSiB ribbon on top (**a**,**d**,**g**) represents these results for 0.2 µF capacitor; (**b**,**e**,**h**) represents these results for 2 µF capacitor and (**c**,**f**,**i**) represents change in inductance results obtained from the RLC meter.

To make the circuit more sensitive to the change in inductance as well as to downselect and shortlist the capacitors for the data acquisition system a bending rig test was performed with 0.2 µF capacitor and 2 µF capacitor with a composite sample with CoSiB ribbon on top. These tests are performed utilising the built data acquisition system and an exemplar of the setup is shown in Figure 9. The obtained results are shown in Figure 10. 

In Figure 10a,d,g corresponds to the change in inductance of a bending rig test with 0.2 µF capacitor and with the 30, 50 and 70 turns inductors, while Figure 10b,e,h corresponds to the change in inductance of a bending rig test with 2 µF capacitor and with 30, 50 and 70 turns inductors. Figure 10c,f,i correspond to the change in inductance with 30, 50 and 70 turns inductors with and the data was being obtained by connecting the inductor to a RLC meter. From the graph it can be observed that using a 2 µF capacitor gives a higher magnitude of change in inductance when compared to the 0.2 µF capacitor. It is necessary to have a higher magnitude of change in inductance to effortlessly detect the damage occurring in composites. It can also be observed that when the 0.2 µF capacitor is used along with the 30 turns inductor and the 70 turns inductor, the measured change in inductance remains the same for both bending rig radiius of 400 and 500 mm. Which proves that 0.2 µF is not sensitive to the applied stress. Similar behaviour is observed while using the 50 turns inductor for the bending rig of 300 mm and 400 mm radii. Considering these sensitivity issues, using a 0.2 µF capacitor would not be able to provide necessary information about the damage occurring in composites.

The change in inductance results of 2 µF capacitor are almost the same as the results obtained using the LCR meter. For example, when the fabricated 30 turn inductor is subjected to a bending test with rig radius 300 mm, a change in inductance of 1.5 µH is observed from the fabricated data acquisition system. When the same experiment is repeated with the RLC meter a change in inductance of 1.15 µH is observed. The difference is 23% when comparing the results from the fabricated data acquisition system and RLC meter. Similar change in inductance is also observed for the other bending rig radii. When the 50-turn inductor was subjected to bending test with the 300 mm radii, the fabricated data acquisition system gave a change in inductance of 2.62 µH. When the same test is repeated with the RLC meter as the data acquisition system, the maximum change in inductance observed was 2.27 µH, which is shown in Figure 10f. This shows a difference of 13% when compared to the change in inductance obtained from the fabricated data acquisition system. When the fabricated 70 turn inductor was subjected to the bending test with the fabricated data acquisition system a maximum change in inductance of 3.5 µH was observed for 300 mm radii. When the same experiment had been repeated with an RLC meter the maximum change in inductance observed was 6.2 µH. The percentage difference between both the results is 56%. From this it can be observed as the number of turns in the inductor increases the results show larger deviation between the fabricated data acquisition system and the RLC meter.

**Figure 11:**
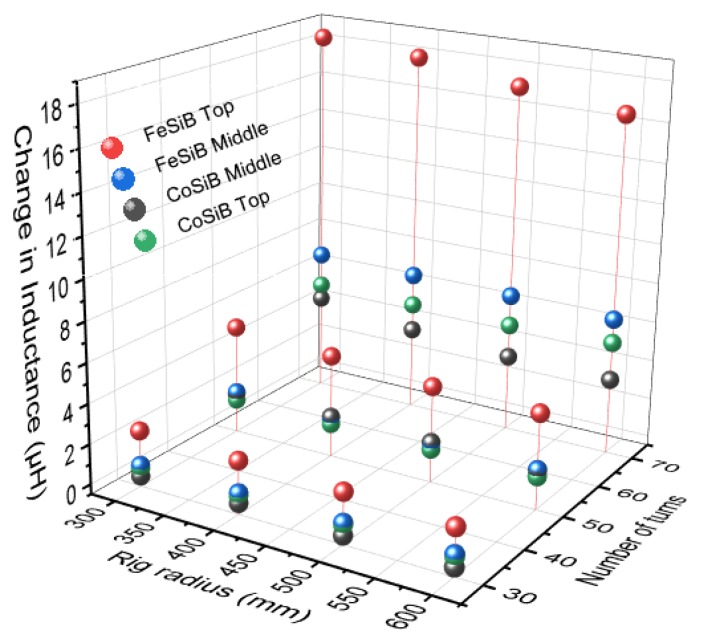
Change in inductance vs number of turns vs rig radius using different actuator materials.

From the previous results it can be observed that as the number of turns increased the change in magnitude of the inductance increased. To detect the damage occurring in composites, it is important to have a system which gives the highest change in magnitude of the inductance when the damage occurs. From the bending test results, it can be concluded that having 2 µF capacitor in the data acquisition system along with the fabricated air cored inductor of 70 turns will be the best combination to detect the damage occurring in the composites. It is also important to select the right magnetostrictive actuator to be coupled along with the sensor for developing a proper structural health monitoring system for composites. As discussed, earlier CoSiB and FeSiB ribbons co-cured along with composites were subjected to bending rig test. The results of the test are shown in Figure 11. From the results, it can be observed that that FeSiB ribbons have a larger magnetostriction response when compared to CoSiB ribbons, as the laminates with FeSiB ribbons produced a higher change in inductance compared to laminates with CoSiB ribbons. The maximum change in inductance was observed when the magnetostrictive ribbons were placed on top of the composite laminate, compared to the ribbons being placed in the middle of the laminate. For example, laminate with FeSiB ribbon on top gave a maximum change of inductance of 18 µH with a 70 turn inductor when tested with a bending rig of 300 mm. Laminate with CoSiB ribbon on top with the same sensor and bending rig gave a maximum change in inductance of 4.2 µH. Whereas when the same test was repeated for laminates containing FeSiB and CoSiB ribbons in the middle in which laminates with FeSiB ribbons gave maximum change in inductance of 6 µH and Laminates with CoSiB gave a change of 3.8 µH. From these results it can be concluded that FeSiB ribbons with actuator on top gave the best change in inductance which will be helpful in achieving structural health monitoring system in composites.

The data shows that actuators placed on the top layer of the composite coupons show a better response. The strain sensitivity resolution of the FeSiB top (30 turns: 0.496, 50 turns: 0.817, and 70 turns: 1.86 μStrain) are higher than that of the CoSiB top (30 turns: 0.047, 50 turns: 0.024, and 70 turns: 0.211 μStrain). From these results it can be concluded that the FeSiB ribbon as an actuator along with the 70 turns coil (has the highest strain sensitivity resolution) would function best as the sensor-actuator for the prototype system in this work. Previous studies using three ribbons *per* composite coupon report strain sensitivity resolution of 17 and 25 μStrain for ribbon spacings of 20 and 10 mm respectively (utilising an inductance pick up coil), which are approximately an order of magnitude larger than these results. For comparison, fibre Bragg gratings have a strain sensitivity resolution of 2000 μStrain [33,34] while piezoelectric sensors are at 150 μStrain [35]. The lower resolution can be mitigated through the use of data procession strategies and the use of multiple sensors in a sparse array. Further lightweighting was attempted by using the designed data acquisition system with a Coilcraft 0.6 µH inductor sensor purchased from Farnell has been subjected to bending rig test with a composite coupon utilising a CoSiB ribbon (top) as an actuator.

**Figure 12:**
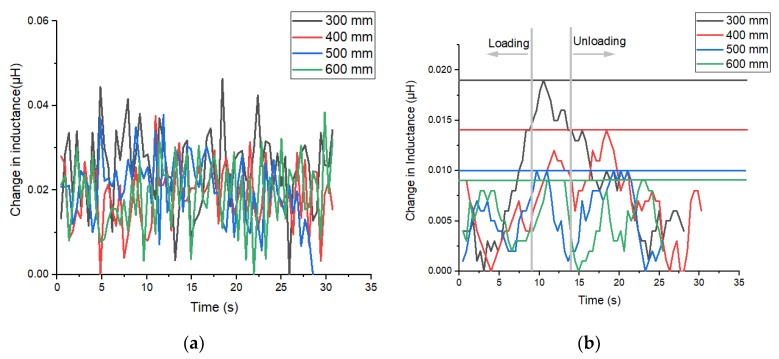
(**a**) Change in inductance with off-the-shelf 0.6 μH Farnall inductor (**b**) Change in inductance by adding shielded inductor in series to the Farnall 0.6 μH inductor where the lines are a guide to the eye.

The results for the change in inductance for each bending rig radius is shown in Figure 12a. The data obtained did not show much change in inductance due to a high noise level. Random noise typically has a peak-to-peak to standard deviation ratio between 6 and 8. The calculated values of the data in Figure 12a reveals ratios of 5.16, 5.17, 4.87, and 4.65 respectively for 600, 500, 400, and 300 mm radii respectively. This suggests that most of the signal would be lost within the noise. To reduce the noise level and to increase the sensitivity of the sensor, a shielded inductor of inductance 115.7 µH was added in series to the 0.6 µH inductor. Figure 12b shows the bending rig test results after the addition of the shielded inductor. The peak-to-peak to standard deviation ratio of these new values are 3.77, 3.92, 3.44, and 3.52 respectively for 600, 500, 400, and 300 mm radii respectively; it is clear from these values that the noise level decreased compared to the case wherein there was no shielded inductor. Accordingly, a visual inspection of both figures show that a change in signal can now be observed. The strain sensitivity resolution in Figure 12b is calculated as 0.01 μStrain, which is approximately an order of magnitude smaller (0.211 μStrain) than the 70-turn coil we had tested earlier when paired with the CoSiB ribbon (top). It is not clear how the strain sensitivity resolution is linked to damage detectability. The results thus although demonstrating the tuneability of the data acquisition system, also highlights additional work that needs to be performed on sensor design and/or acquisition strategies in addition to comparative work that must be done to link the resolution to damage detectability.

## 6. Potential Applications

Magnetostrictive structural health monitoring technique has potential applications in the aerospace industry to detect and monitor cracks in critical aerospace components like airframe, fuselage, etc. This system can also be implemented in the energy sector to monitor and detect damage in oil and gas transportation pipelines, nuclear power plants, and also to detect cracks in wind turbine blades. Magnetostrictive structural health monitoring can also be used to monitor damages occurring in dams, tunnels and mines.

## 7. Limitations and Future Possible Evolution

The current data acquisition has been limited to four inputs which can be improved to higher number of inputs for implementing structural health monitoring on larger scale. Developments in the data acquisition system has to be made to increase the sensitivity and to reduce the noise while using a commercially available air-cored inductor of lower inductance as sensor. 

## 8. Conclusions

In this work, a low-cost data acquisition system that can be used with magnetostrictive systems has been designed around the following principles:

(1) We investigated possible LC combinations to determine damping and frequency responses. We found that the 200 μF capacitor was not suitable as it gave overdamped response for all the inductors. The 2 μF was chosen as the capacitor for the data acquisition system as it gave the best sensitivity results for all sensors in the bending rig test.
(2) Copper-coil inductors (sensors) were fabricated, tested and compared alongside off-the-shelf inductor coils. From the results, the fabricated sensors gave best change in inductance in the bending rig test. The off-the-shelf Farnell inductor gave very noisy results when tested with the RLC meter. However, using a shielded inductor in series with it gave a better result.
(3) The efficacy of FeSiB and CoSiB as magnetostrictive ribbon actuators were investigated utilising the fabricated setup. FeSiB gave the maximum change in inductance as compared to CoSiB in the bending rig tests.

Experimental results show that a high inductance value is required to enable sensing - this disallows most off-the-shelf inductors as their inductance values are too small. However, it is favourable to use low inductance sensors to minimise weight loading. We enable this by connecting a shielded inductor in series.

## Figures and Tables

**Table 1:** Table showing the various sensors fabricated with their corresponding inductance values.

No. of Turns of Copper Coil	Inductance (μH)
30	14.7
50	35.6
70	64.3
